# Prevention of mother-to-child transmission of HIV at the second immunization visit: a cross-sectional study, Burkina Faso

**DOI:** 10.2471/BLT.22.288522

**Published:** 2022-11-01

**Authors:** Béninwendé Leticia Delphine Sakana, Anaïs Mennecier, Paulin Fao, Souleymane Tassembedo, Jean-Pierre Moles, Dramane Kania, Ajani Ousmane Taofiki, Franck Edgar Kadeba, Ibrahima Diallo, Sabrina Eymard-Duvernay, Morgana D’Ottavi, Nicolas Meda, Beatriz Mosqueira, Philippe Van de Perre, Nicolas Nagot, on behalf of the ANRS 12388 PREVENIR-PEV Study Group

**Affiliations:** a Centre Muraz, Bobo-Dioulasso, Burkina Faso.; b INSERM, 60 Rue de Navacelles, 34090 Montpellier, France.; c Département de Santé Publique, Université de Ouagadougou, Ouagadougou, Burkina Faso.

## Abstract

**Objective:**

To evaluate the performance of the cascade of activities for prevention of mother-to-child transmission (PMTCT) of human immunodeficiency virus (HIV) at the second immunization visit in Burkina Faso.

**Methods:**

In a cross-sectional study, we recruited mothers attending the second immunization visit for their infant in 20 health centres of Bobo-Dioulasso city, Burkina Faso over 12 months (2019–2020). We administered a short questionnaire to 14 176 mothers and performed HIV serological tests on mothers who had not been tested in the last 3 months. All mothers were asked about their attendance for antenatal care and HIV rapid testing. HIV-infected mothers were also asked about the timing of their HIV diagnosis, antiretroviral therapy, pre-exposure prophylaxis initiation at birth and infant diagnosis of HIV.

**Findings:**

Of 14 136 respondents, 13 738 (97.2%) had at least one HIV serological test in their lifetime. Of 13 078 mothers who were never tested or were HIV-negative, 12 454 (95.2%) were tested during or after their last pregnancy. Among HIV-infected mothers already aware of their status, 110/111 (99.1%) women were on antiretroviral therapy. Among HIV-exposed infants, 84/101 (83.2%) babies received 6 weeks of antiretroviral prophylaxis at birth and 58/110 (52.7%) had a blood sample collected for early infant diagnosis. Only two mothers received their child’s test results at the time of the second immunization visit. Four mothers were newly diagnosed as HIV-positive during the study.

**Conclusion:**

Collecting data at the second immunization visit, a visit rarely missed by mothers, could be useful for identifying gaps in the PMTCT cascade in settings where mothers are difficult to reach, such as in low-income countries with intermediate or low HIV prevalence.

## Résumé

**Objectif:**

Évaluer les performances de la cascade d'activités liées à la prévention d'une transmission de la mère à l'enfant (PTME) du virus de l'immunodéficience humaine (VIH) lors de la seconde visite de vaccination au Burkina Faso.

**Méthodes:**

Dans le cadre de notre étude transversale, nous avons recruté des mères se rendant à la seconde visite de vaccination de leur nourrisson dans 20 centres de santé de la ville de Bobo-Dioulasso (Burkina Faso) pendant 12 mois (2019–2020). Nous avons soumis un bref questionnaire à 14 176 mères et effectué un test sérologique de dépistage du VIH sur celles n’ayant pas été testées au cours des trois derniers mois. Toutes ont été interrogées sur leur participation aux soins prénatals et aux tests rapides de détection du VIH. Nous avons également demandé aux mères infectées par le VIH des informations sur la date du diagnostic, le traitement antirétroviral, le début de la prophylaxie avant exposition à la naissance et le dépistage du VIH chez le nourrisson.

**Résultats:**

Sur un total de 14 136 participantes, 13 738 (97,2%) ont subi au moins un test sérologique de dépistage du VIH au cours de leur vie. Sur 13 078 mères jamais testées ou séronégatives, 12 454 (95,2%) ont été testées au cours ou à l'issue de leur dernière grossesse. Parmi les mères séropositives connaissant déjà leur statut, 110 sur 111 (99,1%) suivaient un traitement antirétroviral. Et parmi les nourrissons exposés au VIH, 84 sur 101 (83,2%) ont reçu une prophylaxie antirétrovirale durant les 6 semaines suivant leur naissance, tandis qu'un échantillon de sang a été prélevé chez 58 sur 110 (52,7%) afin de poser un diagnostic précoce. Seules deux mères avaient obtenu les résultats des tests de leur enfant au moment de la seconde visite de vaccination. Enfin, quatre mères ont été diagnostiquées séropositives pendant l'étude.

**Conclusion:**

Colliger des données lors de la seconde visite de vaccination, à laquelle les mères sont généralement présentes, pourrait s'avérer utile pour identifier les faiblesses dans la cascade d'activités liées à la PTME dans les régions où il est difficile d'entrer en contact avec les mères, notamment dans les pays à revenu faible où la prévalence du VIH est faible à modérée.

## Resumen

**Objetivo:**

Evaluar el rendimiento de la serie de actividades de prevención de la transmisión maternofilial (PTMF) del virus de la inmunodeficiencia humana (VIH) en la segunda visita de vacunación en Burkina Faso.

**Métodos:**

En un estudio transversal, se reclutaron madres que asistían a la segunda visita de vacunación de su bebé en 20 centros sanitarios de la ciudad de Bobo-Dioulasso, Burkina Faso, durante 12 meses (2019-2020). Se realizó un breve cuestionario a 14 176 madres y se llevaron a cabo pruebas serológicas del VIH a las madres que no se habían hecho la prueba en los últimos 3 meses. Se preguntó a todas las madres sobre su asistencia a la atención prenatal y a la prueba rápida del VIH. También se preguntó a las madres infectadas por el VIH sobre el momento de su diagnóstico de VIH, el tratamiento antirretrovírico, el inicio de la profilaxis previa a la exposición en el momento del nacimiento y el diagnóstico de VIH en el bebé.

**Resultados:**

De 14 136 encuestadas, 13 738 (97,2 %) se hicieron al menos una prueba serológica del VIH a lo largo de su vida. De las 13 078 madres que nunca se habían sometido a la prueba o no estaban infectadas por el VIH, 12 454 (95,2 %) se sometieron a la prueba durante o después de su último embarazo. Entre las madres infectadas por el VIH que ya conocían su estado, 110/111 (99,1 %) mujeres estaban en tratamiento antirretrovírico. Entre los bebés expuestos al VIH, 84/101 (83,2 %) recibieron 6 semanas de profilaxis antirretrovírica al nacer y a 58/110 (52,7 %) se les tomó una muestra de sangre para el diagnóstico temprano del bebé. Solo dos madres recibieron los resultados de las pruebas de sus hijos en el momento de la segunda visita de vacunación. Cuatro madres recibieron un diagnóstico reciente como infectadas por el VIH durante el estudio.

**Conclusión:**

La recopilación de datos en la segunda visita de vacunación, una visita a la que rara vez faltan las madres, podría ser útil para identificar las deficiencias del proceso de PTMF en lugares donde es difícil llegar a las madres, como en los países de bajos ingresos con una prevalencia intermedia o baja del VIH.

## ملخص

**الغرض:**

تقييم أداء سلسلة الأنشطة للوقاية من انتقال فيروس نقص المناعة البشرية من الأم إلى الطفل (PMTCT) في زيارة التحصين الثانية في بوركينا فاصو.

**الطريقة:**

في دراسة مقطعية، قمنا بالاستعانة الأمهات اللاتي حضرن زيارة التحصين الثانية لأطفالهن الرضع في 20 مركزًا صحيًا في مدينة بوبو ديولاسو، بوركينا فاصو على مدى 12 شهرًا (2019-2020). قمنا بطرح استبيان قصير على 14176 من الأمهات، وإجراء اختبار مصلي لفيروس نقص المناعة البشرية للأمهات اللاتي لم يخضعن للاختبار خلال الشهور الثلاثة الأخيرة.. تم توجيه أسئلة لجميع الأمهات عن حضورهن لزيارات لرعاية السابقة للولادة، والاختبار السريع لفيروس نقص المناعة البشرية. كما سألنا الأمهات المصابات بفيروس نقص المناعة البشرية عن توقيت تشخيص إصابتهن بفيروس نقص المناعة البشرية، والعلاج المضاد للفيروسات القهقرية، والبدء في العلاج الوقائي قبل التعرض عند الولادة، وتشخيص فيروس نقص المناعة البشرية لدى الرضع.

**النتائج:**

من بين 14136 مستجيبة، كان لدى 13738 (%97.2) اختبار مصلي واحد على الأقل لفيروس نقص المناعة البشرية في حياتهم. من بين 1378 من الأمهات اللاتي لم يتم اختبارهن، أو كن مصابات بفيروس نقص المناعة البشرية، تم اختبار 12454 (%95.2) أثناء الحمل الأخير، أو بعده. من بين الأمهات المصابات بفيروس نقص المناعة البشرية اللاتي كن يعرفن بالفعل حالتهن، كانت 110/111 (%99.1) من السيدات يتلقين العلاج المضاد للفيروسات القهقرية. من بين الرضع المعرضين لفيروس نقص المناعة البشرية، تلقى 84/101 (%83.2) من الأطفال 6 أسابيع من العلاج الوقائي بمضادات الفيروسات القهقرية عند الولادة، وحصل 58/110 (%52.7) على عينة دم تم جمعها من أجل التشخيص المبكر للرضع. حصلت اثنتان فقط من الأمهات على نتائج اختبار طفليهما في وقت زيارة التحصين الثانية. تم تشخيص أربع أمهات حديثًا على أنهن مصابات بفيروس نقص المناعة البشرية أثناء الدراسة.

**الاستنتاج:**

إن تجميع البيانات في زيارة التحصين الثانية، وهي زيارة نادراً ما تفوتها الأمهات، قد يكون مفيداً في تحديد الثغرات في سلسلة الوقاية من انتقال الفيروس من الأم إلى الطفل في الأوضاع التي يصعب فيها الوصول إلى الأمهات، كما هو الحال في الدول ذات الدخل المنخفض مع الانتشار المتوسط أو المنخفض لفيروس نقص المناعة البشرية.

## 摘要

**目标:**

旨在评估布基纳法索第二次免疫接种中人类免疫缺陷病毒 (HIV) 母婴传播 (PMTCT) 各阶段预防活动的成效。

**方法:**

我们招募在 12 个月的期限内在布基纳法索博博-迪乌拉索市 20 个健康中心为其幼儿孩子进行第二次免疫接种的母亲参加一项横断面研究。我们对 14,176 名母亲进行了简短的问卷调查，并对在过去3个月内未进行检测的母亲进行了艾滋病毒血清检测。同时对于感染 HIV 的母亲,询问其 HIV 确诊时间、采用的抗逆转录病毒治疗、出生时开始的暴露前预防措施及婴儿的 HIV 诊断情况。

**结果:**

在 14 136 名受访者中,13 738 (97.2%) 名受访者至少在人生中某个阶段接受过一次 HIV 血清学检测。在从未接受过 HIV 检测或检测结果为阴性的 13 078 位母亲中,12 454 (95.2%) 位在其最近一次孕期期间或之后接受过检测。在感染 HIV 并且已了解其病情的母亲中,110/111 (99.1%) 位曾接受过抗逆转录病毒治疗。在存在 HIV 感染的婴儿中,84/101 (83.2%) 名婴儿在出生时接受过为期 6 周的抗逆转录病毒治疗,并且 58/110 (52.7%) 名婴儿进行了血样采集,以用于早期婴儿诊断。仅两位母亲在第二次免疫接种时收到了其孩子的检测结果。四位母亲在研究开展期间初次诊断为 HIV 阳性。

**结论:**

在第二次免疫接种(很少有母亲会错过这次接种)时采集数据,有助于确定婴儿母亲在获取诊断方面有困难地区(例如,存在较低或中等程度 HIV 患病率的低收入国家)PMTCT 各阶段之间存在的差距。

## Резюме

**Цель:**

Оценить эффективность комплекса мероприятий по профилактике передачи вируса иммунодефицита человека (ВИЧ) от матери ребенку (ППМР), осуществляемых во время второго визита для проведения вакцинации в Буркина-Фасо.

**Методы:**

Для участия в кросс-секционном исследовании были отобраны матери, которые пришли на второй визит для проведения вакцинации своего ребенка в 20 медицинских центрах города Бобо-Диуласо, Буркина-Фасо, в течение 12 месяцев (2019–2020 гг.). Среди 14 176 матерей было проведено краткое анкетирование, а среди матерей, которые не были протестированы в течение последних 3 месяцев, также было проведено серологическое тестирование на ВИЧ. Всем матерям задавали вопросы о том, посещали ли они дородовое наблюдение и проходили ли экспресс-тестирование на ВИЧ. ВИЧ-инфицированных матерей также спрашивали о времени постановки диагноза ВИЧ, проведении антиретровирусной терапии, начале доконтактной профилактики при рождении и диагностике ВИЧ у детей грудного возраста.

**Результаты:**

Из 14 136 респондентов 13 738 (97,2%) прошли хотя бы одно серологическое исследование на ВИЧ в течение жизни. Из 13 078 матерей, которые никогда не проходили тестирование или были ВИЧ-отрицательными, 12 454 (95,2%) прошли тестирование во время или после последней беременности. Среди ВИЧ-инфицированных матерей, уже знавших о своем статусе, 110 из 111 женщин (99,1%) проходили антиретровирусную терапию. Среди ВИЧ-инфицированных детей грудного возраста 84 из 101 ребенка (83,2%) провели 6-недельную антиретровирусную профилактику при рождении, а у 58 из 110 младенцев (52,7%) был взят образец крови для ранней диагностики. Только две матери получили результаты анализов своего ребенка во время второго визита для проведения вакцинации. В ходе исследования у четырех матерей был впервые установлен диагноз ВИЧ-инфекции.

**Вывод:**

Сбор данных во время второго визита для проведения вакцинации, который редко пропускается матерями, может быть полезен для выявления пробелов в комплексе мероприятий по профилактике ППМР в условиях, когда сложно связаться с матерями, например в странах с низким уровнем дохода и средней или низкой распространенностью ВИЧ.

## Introduction

Despite progress in preventing new paediatric human immunodeficiency virus (HIV) infections, the goal of eliminating mother-to-child transmission remains unattained.^1^ According to an estimate from the United Nations Children’s Fund, approximately 150 000 children worldwide were newly infected with HIV in 2020.^2^ Furthermore, children living with HIV are frequently underdiagnosed.^3^

Successive international guidelines on the prevention of mother-to-child transmission (PMTCT) have been issued over the last decade, with the goal of eliminating mother-to-child transmission of HIV. HIV testing for pregnant women and all HIV-exposed infants within 6 weeks after birth has been recommended for over a decade.^4^^,^^5^ In 2013, the World Health Organization (WHO) recommended the test-and-treat strategy (called option B+). The strategy includes lifelong antiretroviral therapy for pregnant and breastfeeding mothers, and 4–6 weeks of single drug pre-exposure prophylaxis for HIV-exposed infants.^6^ In 2016, dual-drug pre-exposure prophylaxis was recommended for high-risk children during the first 6 weeks of life. The recommended duration of treatment was increased to 12 weeks for breastfed children at high risk of contracting HIV.^7^

The PMTCT cascade is a succession of key activities and associated indicators for the overall period in which children are at risk of HIV infection: that is, from conception to the cessation of breastfeeding for HIV-exposed uninfected children. This series of steps facilitates the identification of the gaps that need to be addressed, and influences decision-making. Nevertheless, the lack of reliable data is often an obstacle to meaningful analysis and can impede health-care professionals from making decisions that are needed to decrease mother-to-child transmission of HIV.^8^ This lack of reliability occurs because data from mothers who are missed out of the PMTCT programme are not accounted for, and because the quality of the data collected depends on the performance of the facilities. 

In Burkina Faso, the HIV prevalence among 11 627 tested pregnant women (aged between 15 and 49 years) was 1.3% (95% confidence interval, CI: 1.07–1.48) in 2018. In Bobo-Dioulasso, the second largest city in the country with an urban population of 900 000, the prevalence of HIV was 1.7% (95% CI: 1.10–2.75). Young women (5813 women aged 15–24 years old) were less affected, with a 0.8% (95% CI: 0.63–1.12) and 0.6% (95% CI: 0.18–1.7) prevalence of HIV in Burkina Faso and in Bobo-Dioulasso, respectively.^9^ According to the national guidelines, HIV testing is offered to pregnant women every 3 months.^10^ HIV-exposed infants should systematically receive 6 weeks of nevirapine for HIV prophylaxis from birth. Regardless of the mother’s HIV status, all mother–child pairs should attend the 42nd-day healthy baby visit, during which a blood sample is taken from HIV-exposed infants for early diagnosis of infection. The second immunization visit – a visit that is detached from the PMTCT programme but integrated into health and social promotion centres – is performed when the baby is 2 months old. In addition to visits to the centres, some health and social promotion centres organize mobile outreach immunization activities (in private health facilities or outdoor places). In 2020, these mobile visits represented 2336 (9.5%) of the 24 590 second immunization visits conducted by the health and social promotion centres involved in the study (data provided directly by the centres).

The objective of this study was to assess the effectiveness of the PMTCT cascade up to 2 months postpartum in Bobo-Dioulasso, Burkina Faso. To bypass recruitment bias in studies of the PMTCT cascade, we took advantage of the second infant immunization visit – a visit that is almost never missed by mothers and their infants outside the PMTCT programme.^11^

## Methods

### Study design 

We conducted this cross-sectional study at 20 health and social promotion centres in the urban area of Bobo-Dioulasso from 4 December 2019 to 4 December 2020. Outreach visits were not included. The study comprised component 1 of the Elimination of Paediatric HIV-1 Infection: Evaluation of the Prevention Programme and Rescue Intervention Based on the Expanded Programme on Immunization (ANRS 12388 PREVENIR-PEV) study; trial registration number: NCT03869944. The ANRS 12388 PREVENIR-PEV study protocol was approved by the ethics committee for health research and the health authority of Burkina Faso.

### Data collection 

We enrolled mothers aged 15 years or older who attended the second immunization visit for immunization of their 5–16-week-old infants. The staff of the health and social promotion centres administered a short questionnaire to mothers after obtaining opt-out consent from mothers and before the infants were vaccinated (the questionnaire is available in the data repository).^12^ The questions collected only basic information to minimize bias associated with self-reporting, as well as to avoid discouraging mothers from participating in the study if the questionnaire were too cumbersome. The questionnaire concerned mothers’ activities related to PMTCT during the current pregnancy. All mothers were asked about their attendance for antenatal care and HIV rapid testing. HIV-infected mothers were in addition asked about the timing of their HIV diagnosis, whether they were on antiretroviral therapy (ART), whether their infant had received pre-exposure prophylaxis at birth, and their infant’s diagnosis of HIV at birth. 

Staff checked the newborns’ health record booklets for information about the mothers’ HIV status. This strategy avoided considering mothers as newly diagnosed with HIV when they were already aware of their status. Indeed, some mothers prefer not to reveal their positive status to health-care providers for fear of stigmatization, as observed in another study in Africa.^13^ An HIV rapid test was offered to all mothers with no relevant documented test results within the last 3 months. A mother was considered HIV-infected if she had two positive HIV rapid tests (Alere Determine HIV-1/2 SD, Abbott Laboratories, Chicago, United States of America), confirmed by Bioline HIV 1/2 (Abbott Laboratories, Chicago, USA) or OnSite HIV 1/2 Ab Plus Combo rapid test (CTK Biotech, Poway, USA), as per the national algorithm.

The mothers’ questionnaires were formatted as electronic case report forms using the REDCap software (Vanderbilt University, Nashville, USA) and recorded by site staff on electronic tablets. The data collected on the tablets were synchronized daily with the web server. We carried out on-site and centralized monitoring to increase data quality and prevent errors and possible oversights by staff.

In addition, we retrieved the data collected by the district regarding the monthly second immunization visit attendance in the 20 health and social promotion centres in the study.

### Data analysis

We included all enrolled participants in the analysis. We present descriptive summary statistics for outcome variables of interest using percentages and 95% confidence intervals (CIs). We omitted missing data from the denominators for percentage calculations. Specific key indicators were stratified according to the maternal age. We calculated relative risks (RRs) with 95% CIs to evaluate if certain age groups were associated with lower rates of antenatal care attendance and missed opportunities for HIV rapid testing, using the over-24-year age group as the reference group.^14^ We performed statistical analyses using Stata version 16.1 (Stata Corp., College Station, USA).

## Results

### Study population

**Fig. 1:**
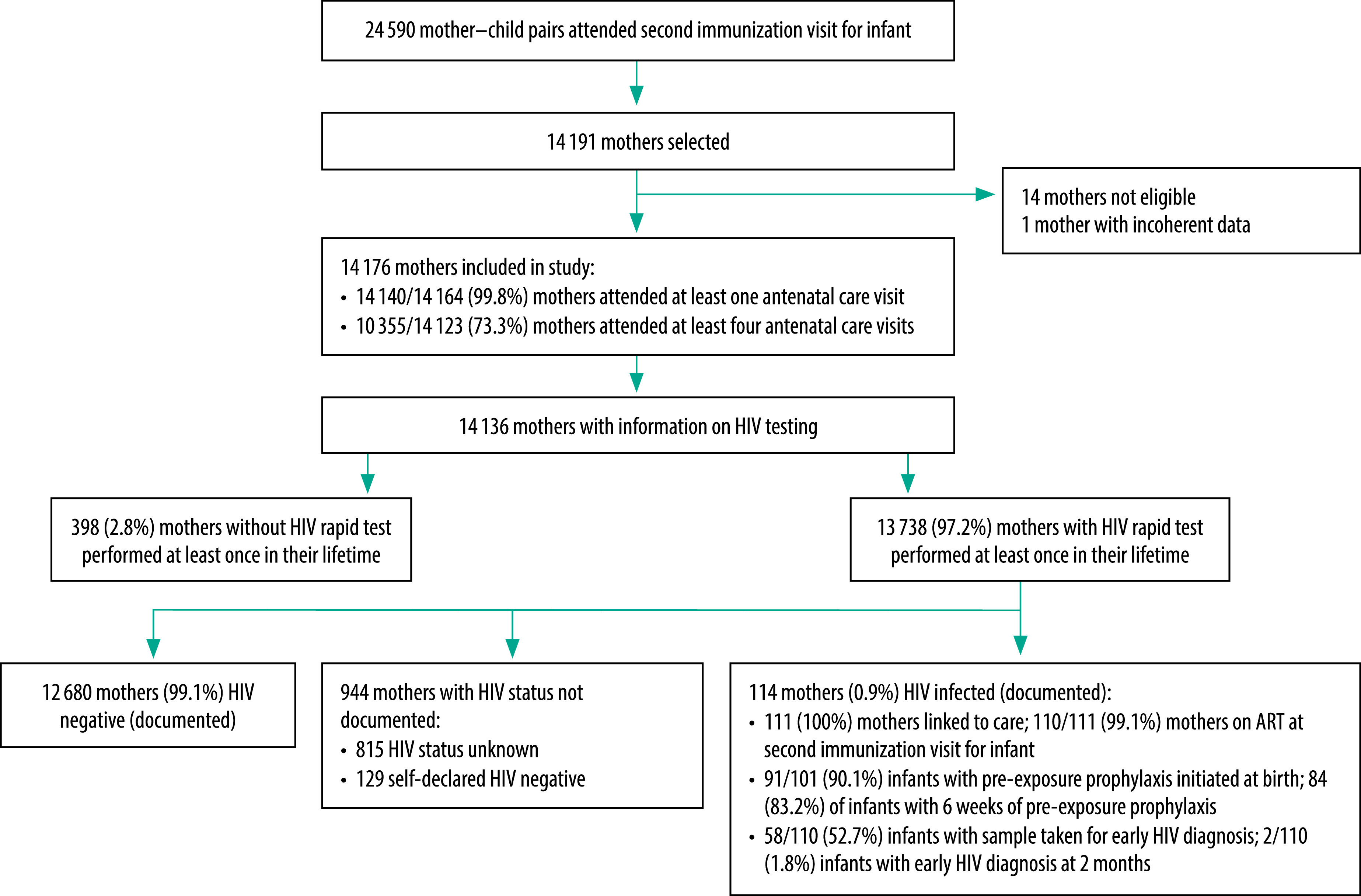
Flowchart of the study of prevention of mother-to-child transmission of human immunodeficiency virus in Bobo-Dioulasso city, Burkina Faso, 2019–2020

ART: antiretroviral treatment; HIV: human immunodeficiency virus.

In 2020, 24 590 mother–child pairs participated in the second immunization visit at the 20 participating health and social promotion centres, according to district data. Between 4 December 2019 and 4 December 2020, a total of 14 191 mothers were screened, and 14 176 were enrolled in this study (Fig. 1). The median age of the enrolled mothers was 25.9 years (interquartile range: 21.3–31.0 years). Among the enrolled mothers, 14 140 (99.8%; 95% CI: 99.8–99.9; 12 missing answers) had attended at least one antenatal care visit during their last pregnancy and 10 355 (73.3%; 95% CI: 72.6–74.0; 53 missing answers) had attended four or more antenatal care visits.

### HIV test status

Among the 14 176 mother–child pairs, 398 mothers declared they had never been tested (2.8%; 95% CI: 2.5–3.1) and 13 738 mothers had had at least one HIV rapid test in their lifetime (97.2%; 95% CI: 96.9–97.5; 40 missing answers). Overall, among all participants, 235 mothers (1.7%) reported that they had never been offered HIV testing, 69 mothers had refused testing and 94 mothers had accepted testing but ultimately had not been tested.

Among the mothers who had been tested, the last documented result was HIV-negative for 12 680 mothers (corresponding to 99.1% of 12 794 participants with a clear positive or negative HIV status) and HIV-positive for 114 mothers (0.9%). Of the 398 mothers never tested for HIV, 391 mothers (98.7%; 2 missing answers) had attended at least one antenatal care visit during their last pregnancy and 206 mothers (52.4%; 5 missing answers) had attended four or more antenatal care visits. 

A total of 12 454 mothers had been tested for HIV during or after the last pregnancy (up to the second immunization visit). This figure represents 98.3% (95% CI: 98.1–98.5; 9 missing answers) of the 12 680 HIV-negative mothers, or more broadly 95.2% of the 13 078 mothers never tested or with negative status. Among the mothers tested during or after the last pregnancy, 8263 mothers (65.7%; 95% CI: 64.9–66.5; 106 missing answers) had been tested in the last 3 months, in line with national recommendations.

### Awareness of HIV status 

Among the HIV-positive participants, 114 out of 118 mothers (96.6%) were already aware of their HIV infection. Of these 114 mothers, 76 (68.1%; 95% CI: 59.8–77.1; 4 missing answers) were diagnosed before their last pregnancy and 35 (31.5%) were diagnosed during or after their last pregnancy (no information for 3 mothers).

Of the 111 mothers who provided information concerning ART, all women reported that they had been offered ART; we therefore considered them as linked to care. All but one of these mothers (110 mothers; 99.1%) were on ART at the time of the second immunization visit. Nevirapine prophylaxis was initiated at birth in 91 infants (90.1%; 95% CI: 84.3–95.9; 13 missing answers) and 84 of these HIV-exposed babies (83.2%; 95% CI: 75.9–90.5; 13 missing answers) received prophylaxis for the entire recommended 6-week period. According to mothers, blood samples were collected at the 42nd day visit for early infant diagnosis for 58 babies (52.7%; 95% CI: 43.4–62.1; 3 missing answers and one mother did not know if samples had been collected or not). However, only two mothers were aware of the test result at the time of second immunization visit (1.8%; 4 missing answers).

Overall, 5606 mothers did not have a documented HIV test performed in the last 3 months and were tested for HIV. These women included 379 mothers who had never been tested before (19 of 398 mothers never tested before did not receive a test during the study for various reasons). Four mothers were diagnosed as HIV-positive. Among these, one mother had a negative HIV test result during the last pregnancy and two mothers had never been tested before (one mother with no information).

### HIV status by age

When compared with the 8528 mothers over the age of 24 years, the 1277 adolescent mothers (19 years or younger) were significantly more likely to have not attended any antenatal care visits (RR: 3.34; 95% CI: 1.35–8.26); not attended four or more antenatal care visits (RR: 1.33; 95% CI: 1.22–1.45); and never been tested for HIV (RR: 1.44; 95% CI: 1.06–1.96). Young mothers (4373 aged 20–24 years) also had a significantly higher risk of not having attended at least four antenatal care visits than had older mothers (RR: 1.16; 95% CI: 1.10–1.24) (Table 1).

**Table 1:** Antenatal care visits and time since last human immunodeficiency virus test by mother’s age group in Bobo-Dioulasso city, Burkina Faso, 2019–2020

Variable	Age 15–19 years (*n* = 1277)				Age 20–24 years (*n* = 4373)				Age > 24 years (*n* = 8528)^a^	
	Total no. of mothers responding	No. (%) of mothers	RR (95% CI)		Total no. of mothers responding	No. (%) of mothers	RR (95% CI)		Total no. of mothers responding	No. (%) of mothers
**All mothers**										
Attended no antenatal care visits	1274	7 (0.5)	3.34 (1.35–8.26)		4366	3 (0.1)	0.42 (0.12–1.45)		8512	14 (0.2)
Attended < 4 antenatal care visits	1272	417 (32.7)	1.33 (1.22–1.45)		4352	1252 (28.8)	1.16 (1.10–1.24)		8487	2096^b^ (24.7)
Never tested for HIV	1270	48 (3.8)	1.44 (1.06–1.96)		4359	128 (2.9)	1.12 (0.91–1.39)		8495	222 (2.6)
**HIV-negative mothers**										
Not tested for HIV during or after the last pregnancy	1133	15 (1.3)	0.70 (0.41–1.20)		3923	60 (1.5)	0.82 (0.61–1.10)		7603	142 (1.9)
Not tested for HIV in the last 3 months	1128	381 (33.8)	0.98 (0.90–1.07)		3899	1331 (34.1)	0.99 (0.94–1.04)		7535	2597^c^ (34.5)

CI: confidence interval; HIV: human immunodeficiency virus; RR: risk ratio.

^a^ Reference group.

^b^ Age was missing for three mothers who answered the questions.

^c^ Age was missing for two mothers who answered the questions.

Notes: A total of 14 176 mother–child pairs were included in the study. *n* is total number of mothers enrolled in each age group. We compared mothers’ risk of deviation from the prevention of mother-to-child transmission of HIV programme between age groups using relative risk and their 95% CIs.

Of the 118 HIV-infected mothers, 108 (including three women who were newly diagnosed) were older than 24 years, eight women (including one woman newly diagnosed) were between 20 and 24 years of age, and two women were 19 years or younger.

## Discussion

According to the data recorded in this survey, all the WHO process indicators for achieving elimination of mother-to-child transmission^15^ were reached. We found that 99.8% of women had attended at least one antenatal care visit, 95.2% of mothers had been tested for HIV during pregnancy (or breastfeeding up to the second immunization visit), and 99.1% of HIV-positive mothers were on ART. Improvements in PMTCT facilities and organization in Burkina Faso^16^ and in pregnant women’s adherence to the PMTCT programme over the last decade^17^ have contributed to these impressive results.

The WHO impact indicators^15^ correspond to different rates of new paediatric HIV infections. These indicators are only relevant if the country’s health system allows for effective and exhaustive diagnosis of infants with HIV. The main gap identified during the study was the low proportion of HIV-exposed infants who had their sample collected for diagnosis before 2 months of age (52.7%) and the much lower proportion for those with results available at 2 months of age (1.8%). Our figure is far below the 10% rate of early infant diagnosis for Burkina Faso reported by the Joint United Nations Programme on HIV/AIDS (UNAIDS).^18^ This difference may be explained by the choice of denominator (an estimate in the case of the UNAIDS data)^19^ and numerator (number of samples tested or results disclosed to mothers). Either result (1.8% or 10%) remains far from the UNAIDS interim target of 95% by 2025.^20^

We identified two critical bottlenecks in testing infants for HIV in this study; first, samples were not always collected; and second, turnaround times for test results were prolonged. Our data show an improvement compared with figures in the 2017 annual report for Burkina Faso, in which only 902 (36%) of the 2476 HIV-exposed infants had a sample taken for early infant diagnosis of HIV.^21^ However, increasing the collection of dried blood samples in PMTCT settings is still needed. Long turnaround times can be attributed to the frequent shortages of reagents, as described some years ago in the central region of Burkina Faso,^16^ and to the complex system for nucleic acid testing. The transfer of samples and results between the health and social promotion centres, referral health centres and central laboratory could be optimized. Although infants may receive their results later, early infant diagnosis is defined as a test received within the first 2 months of life so that treatment can be started as soon as possible.^19^ However, with samples collected at 42 days of age for infants, and the organization of HIV PCR testing in a central laboratory, the 2-month date is easily missed. Point-of-care early infant diagnosis reduces the lag in delivery of the results and consequently promotes early initiation of ART.^22^^,^^23^

Although most women participating in the study attended antenatal care visits, adolescents (15–19-year-olds) and young mothers (20–24-year-olds), according to the UNAIDS definition,^14^ were significantly less compliant than older mothers. These findings are consistent with those of previous studies in Africa.^24^^,^^25^ This result may be attributed to fear of stigmatization in the context of illegitimate pregnancies and inadequate knowledge and misconceptions about antenatal care services.^25^ Low compliance with antenatal care visits has implications for obstetric outcomes as well as HIV prevention, with lower testing rates, as demonstrated for adolescent mothers in this study. Pregnancy is a frequent entry point for accessing HIV prevention, especially for adolescent girls and young women, who are considered priority target populations.^14^

Over half of the mothers who attended the second immunization visit were asked to participate in the study (14 191 out of 24 590 mothers; 57.7%). Researchers were in close contact with the health centre staff and identified a variety of reasons for the low recruitment to the study. These challenges included: difficulty for staff in deploying electronic tablets for data collection; overburden of some staff in addition to their daily activities; staff turnover and difficulty in training new staff; and low pay or late payment of wages (as reported by some staff) reducing the incentive to work. We cannot evaluate the representativeness of the study population as sociodemographic or health data for the included mother–child pairs were not recorded. The HIV prevalence in our study (0.9%) was lower compared with that observed in the 2018 Burkina Faso serosurveillance report (1.7%, 18/1034 samples for the Bobo-Dioulasso urban area), although the population was similar (pregnant women with a median age of 25 years).^9^ The non-inclusion of mothers coming for the second immunization visit in our study was not related to the mother’s HIV status, but rather to the recruitment challenges described above. 

This study has some limitations. Most of the questions concerned past events and were susceptible to recall bias, even though the time that had elapsed since the events in question was 6 months or less. The study was conducted in the urban area of a single city, Bobo-Dioulasso. PMTCT implementation is context-dependent, and challenges in other cities and rural areas may differ from those observed in the present study. Another study that is ongoing in a different city in Burkina Faso and in Zambia^26^ will help to validate the study methods in countries with high HIV prevalence. An additional limitation is that infants who died before reaching 2 months of life were not tracked. No data were collected on why the rate of early infant diagnosis was so low; however, an ongoing qualitative study will likely address this concern. Finally, the study assessed the PMTCT cascade up to 2 months postpartum and not until the end of HIV exposure via breastfeeding (breastfeeding being prolonged well beyond 2 months for the majority of infants).

We acknowledge that no single method can fully measure the effectiveness of a programme. WHO considers statistical modelling, surveys and analysis of routine programmes to be complementary.^27^ Recommendations are focused on a group of priority countries in which high HIV prevalence may facilitate implementation, and Burkina Faso is not included.^28^ In the context of low or intermediate HIV prevalence, especially in a low-income country, routine prospective national programme monitoring can be difficult to set up.

Regular assessment of the PMTCT cascade, measured among the entire population, is necessary to analyse the improvements and gaps in a constantly changing scientific, political, economic and public health context.^15^^,^^29^ We propose that the second visit for infant immunization provides this opportunity and can be implemented by national programmes in low-income countries with low to intermediate HIV prevalence.

WHO recommends a shift from aggregate to individual-level data collection.^30^ The implementation of electronic medical records, as advocated by WHO, is not feasible for low-income countries with a low to intermediate HIV prevalence. Nevertheless, the integration of a short questionnaire for mothers during routine second immunization visit activities is highly feasible. In our study, this activity was well accepted by staff and patients. By replacing the aggregate data collected by facilities for annual activities with our strategy on a continuous basis, countries would obtain several benefits. First, they would benefit from a common data source for different uses: patient care, programme management and programme monitoring.^30^ Second, countries could benefit from obtaining denominators for all PMTCT indicators. Third, countries would avoid the risk of overestimating indicators. Although we conducted no cost analysis, the main costs are attributed to staff compensation and appear to be affordable for national programmes.

Although Burkina Faso is not a country with a high HIV prevalence, mother-to-child transmission is a concern, with UNAIDS estimating a final transmission rate of 15% in 2020.^18^ Our strategy, a quick questionnaire among mothers during the second immunization visit, is simple to apply and centred on the people who benefit. We believe that routine implementation of the strategy is appropriate for assessing the effectiveness of the PMTCT cascade in low-income countries with low HIV prevalence, where mothers are difficult to reach.
